# Nine‐Year Follow‐Up After M‐TEER for Secondary Mitral Regurgitation in a Patient With Cardiogenic Shock

**DOI:** 10.1002/ccd.70678

**Published:** 2026-05-27

**Authors:** Tobias Reithmayer, Thomas J. Stocker, Ludwig T. Weckbach, Julia Novotny, Philipp M. Doldi, Hannah Kempton, Steffen Massberg, Michael Näbauer, Jörg Hausleiter, Lukas Stolz

**Affiliations:** ^1^ Medizinische Klinik und Poliklinik I Klinikum der Universität München Munich Germany; ^2^ German Center for Cardiovascular Research (DZHK) Partner Site Munich Heart Alliance Munich Germany

**Keywords:** cardiogenic shock, dilated cardiomyopathy, long‐term follow‐up, MitraClip, mitral regurgitation, M‐TEER

## Abstract

**Background:**

Transcatheter edge‐to‐edge repair (TEER) has become an established therapeutic option for patients with severe secondary mitral regurgitation (SMR). While randomized trials and registry data have reported outcomes up to 5 years, longer follow‐up data remain scarce.

**Case Summary:**

We report the case of a 48‐year‐old male patient who was admitted to our hospital with cardiogenic shock, first diagnosis of dilated cardiomyopathy and severe ventricular SMR in August 2016. With the use of mechanical circulatory support, the patient underwent urgent M‐TEER with implantation of one MitraClip device, reducing MR from severe to mild‐to‐moderate. At 9‐year follow‐up in 2025 and consequent optimization of guideline directed medical therapy, the patient remains clinically stable, with sustained MR reduction and good device function.

**Discussion:**

This case highlights the durability of M‐TEER even in a critically ill patient initially presenting with cardiogenic shock and multiorgan failure. To our knowledge, this represents one of the longest follow‐up observations after M‐TEER, supporting its role as a durable treatment strategy in high‐risk patients.

**Conclusions:**

M‐TEER can achieve durable long‐term MR reduction and symptomatic benefit.

## 1 History of Presentation

A 48‐year‐old man was admitted to our hospital in August 2016 with cardiogenic shock due to dilated cardiomyopathy with severely reduced left ventricular (LV) function. He had initially presented 1 week earlier to the emergency department of a referring hospital with progressive dyspnea corresponding to NYHA functional class IV and recurrent palpitations. At admission, the patient developed tachycardic atrial fibrillation, which deteriorated into cardiogenic shock requiring intubation and invasive mechanical ventilation. During attempted cardioversion with intravenous amiodarone, he suffered two episodes of cardiac arrest, each requiring approximately 10 min of resuscitation.

On arrival in our intensive care unit, the patient was critically ill with respiratory failure, high catecholamine demand, and multiorgan dysfunction. Laboratory investigations showed an estimated glomerular filtration rate (eGFR) of 25 mL/min/1.73 m^2^, bilirubin 10.4 mg/dL, aspartate aminotransferase 7791 U/l, alanine aminotransferase 2769 U/l consistent with acute renal and hepatic failure in the setting of cardiogenic shock. Echocardiography revealed a severely reduced LVEF of 25%, a markedly dilated LV, and severe functional mitral regurgitation with pulmonary congestion. The patient was in persistent atrial fibrillation with rapid ventricular response, and laboratory tests demonstrated thrombocytopenia and elevated inflammatory markers.

A computed tomography (CT) pulmonary angiogram and aortogram excluded pulmonary embolism and aortic dissection. Coronary angiography showed no relevant coronary artery disease. Given the combination of severe MR, left‐sided congestion, and hemodynamic instability, an Impella CP microaxial flow‐pump (Abiomed, Danvers, MA) was implanted for circulatory support. Large bilateral pleural effusions were drained, which improved respiratory mechanics, and continuous hemodiafiltration was initiated for acute renal failure.

## 2 Past Medical History

Prior to the index hospitalization in August 2016, the patient's medical history was significant for chronic alcohol use disorder, with a daily consumption of approximately eight standard drinks, and a panic disorder. There was no documented history of systemic hypertension, diabetes mellitus, chronic obstructive pulmonary disease, chronic kidney disease, or structural heart disease. He had never undergone cardiac surgery, valvular intervention, or device implantation. At the time of admission, the diagnosis of dilated cardiomyopathy was established for the first time in the setting of cardiogenic shock. No prior neurologic events such as stroke or transient ischemic attack were reported.

## 3 Investigations

**Figure 1:**
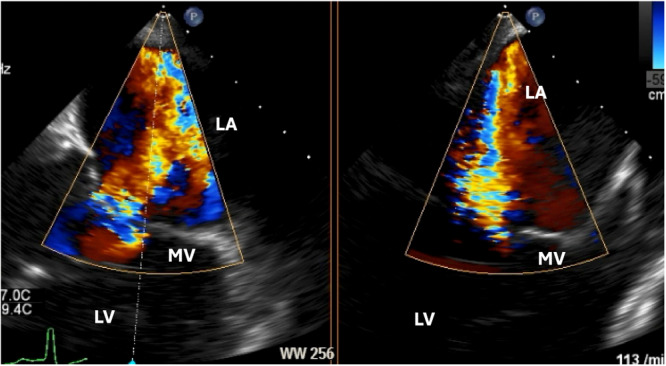
Baseline transesophageal echocardiography. Figure 1 shows transesophageal imaging of severe secondary ventricular mitral regurgitation. Imaging quality was impaired due to artefacts of the implanted Impella CP pump. [Color figure can be viewed at wileyonlinelibrary.com]

**Figure 2:**
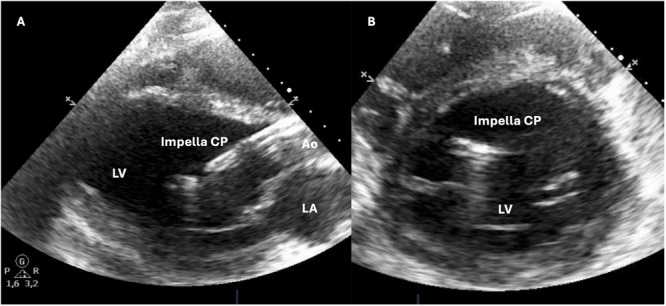
Mechanical circulatory support using an Impella CP pump. Figure 2 illustrates the implanted Impella CP pump in a parasternal long‐axis (A) and a short axis view (B). The left ventricle showed severe dilation and reduced function.

Transthoracic and transesophageal echocardiographic evaluation confirmed the diagnosis of severe SMR (effective regurgitant orifice area 0.31 cm^2^, regurgitant volume 41 mL, Figure 1). The mitral valve showed a ventricular‐secondary MR phenotype, owing to severe left ventricular dilation, with severely reduced function (LVEF 25%, LVEDV 230 mL, LVESV 173 mL), and a markedly dilated left ventricle with preserved RV function (TAPSE 20 mm). There was no evidence of other valvular pathologies. The overall imaging quality was reduced due to artefacts of the implanted Impella CP pump (Figure 2).

## 4 Management

**Figure 3:**
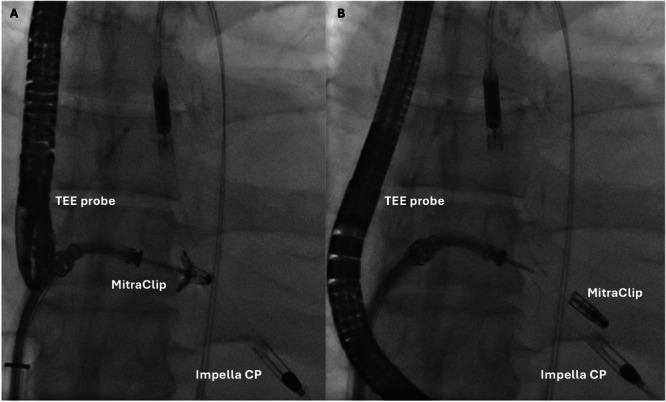
Intraprocedural fluoroscopic imaging. Figure 3A,B illustrates implantation of one MitraClip in the patient with an already implanted Impella CP pump. In panel A the device is in an open position and still detached to the delivery catheter while in panel B the device is closed and released.

**Figure 4:**
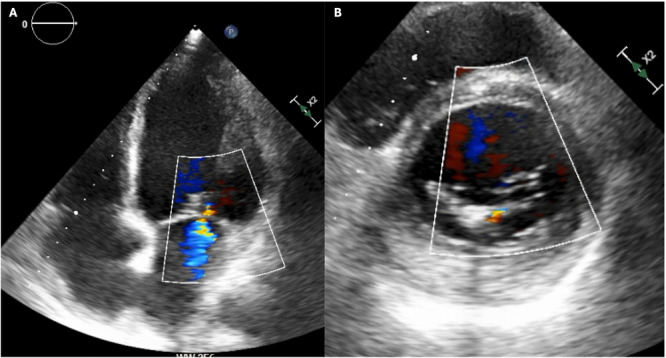
Postprocedural mitral regurgitation. Figure 3A,B shows mild‐to‐moderate residual MR lateral of the centrally implanted MitraClip device. [Color figure can be viewed at wileyonlinelibrary.com]

Given the patient's high surgical risk (EuroSCORE I 30.7%, EuroSCORE II 26.3%, STS score 31.9%) and critical condition with multiorgan dysfunction, the interdisciplinary heart team opted for an urgent interventional treatment approach of SMR. The patient was deemed anatomically suitable for transcatheter edge‐to‐edge mitral valve repair (M‐TEER). A single MitraClip NT device was implanted in a central A2‐P2 position, resulting in a significant reduction of mitral regurgitation from grade severe to mild‐to‐moderate (Figures 3 and 4). The postprocedural mean mitral valve (MV) inflow gradient was 4 mmHg.

Hemodynamic support with an Impella CP pump was maintained during the intervention and gradually weaned after stabilization. Within the further course, the patient was extubated and discharged from ICU to a rehabilitation clinic after 26 days. Guideline directed medical therapy was carefully initiated after weaning from medical and mechanical circulatory support.

At discharge, echocardiography revealed mild residual SMR with stable device position, a mean MV inflow gradient of 4 mmHg and no evidence of device‐related complications.

## 5 Follow‐up

The patient regularly attended our outpatient clinic for scheduled follow‐up visits (Table 1). At 1‐month follow‐up, he already presented with markedly improved functional capacity (NYHA class II). Echocardiography showed recovery of LVEF to 45% with stable device position and a residual mild‐to‐moderate SMR. There was no evidence of peripheral edema, jugular vein distension, pleural effusion, or ascites. Renal function had improved to an eGFR of 59 mL/min.

**Table 1:** Clinical, laboratory and echocardiographic changes over the course of 9‐year follow‐up.

	Baseline	6 months	1 year	2 years	3 years	4 years	5 years	6 years	7 years	8 years	9 years
**A. Heart Failure symptoms**											
*NYHA*	IV	II	II	II	II	I	I	I	I	I	I
*6MWD, m*	0	na	na	389	406	435	442	480	516	552	567
*MLHFQ, points*	na	na	na	31	34	3	1	5	0	7	17
**B. Laboratory data**											
*NT‐proBNP, pg/mL*	na	84	127	121	124	144	92	82	72	107	123
*eGFR, mL/min/1.73 m* ^*2*^	25	87	82	87	83	87	89	85	96	100	80
*Bilirubin, mg/dL*	10.4	0.4	na	na	0.5	0.5	0.4	0.3	0.4	0.4	0.6
**C. Echocardiography**											
MR severity	4+	1(−2)+	1(−2)+	1+	1+	1+	1+	1+	1+	1+	1+
LVEF, %	15	41	45	67	64	60	61	62	59	57	58
**D. Medication**											
*RAS‐I, mg/d (Candesartan)*	none	1.25	4	4	4	8	8	8	8	8	8
*Beta‐blocker, mg/d (Bisoprolol)*	none	10	2.5	5	2.5	2.5	5	5	2.5	2.5	2.5
*MRA, mg/d*	none	none	none	none	none	none	none	none	none	none	none
*SGLT2i, mg/d (Dapagliflozin)*	none	none	none	none	none	none	none	none	10	10	10

Abbreviations: eGFR, estimated glomerular filtration rate; LVEF, left‐ventricular ejection fraction; MLHFQ, Minnesota living with heart failure questionnaire; MR, mitral regurgitation; NT‐proBNP, N‐terminal pro brain natriuretic peptide; NYHA, New York Heart Association; 6MWD, 6‐min walking test distance.

At 6‐month and 1‐year follow‐up, the patient remained in NYHA class II without clinical signs of congestion. NT‐proBNP had decreased to 84 pg/mL, and LVEF improved to 48%. Echocardiography revealed a residual mild mitral regurgitation. At 1‐year, renal function further recovered (eGFR 82 mL/min/1.73 m^2^) with stable NT‐proBNP levels (127 pg/mL) and persistent mild MR. With improving hemodynamics after successful TEER, a gradual uptitration of GDMT became possible.

From the second year onwards, the patient reported a continuous improvement in exercise tolerance. At 2‐year follow‐up, the 6‐min walking distance (6MWD) was 389 m and the Minnesota Living with Heart Failure Questionnaire (MLHFQ) score 31 points. Echocardiography revealed normalization of LVEF (67%) and sustained mild MR. At 3‐year follow‐up, functional capacity was stable (6MWD 406 m, MLHFQ 34 points), LVEF 64%, and there was no evidence of congestion. Laboratory tests indicated normalized hepatic function.

At 4 years, the patient was in NYHA class I with improved exercise tolerance (6MWD 435 m, MLHFQ 3), LVEF 60%, low NT‐proBNP (144 pg/mL), and stable mild MR. From years 5–7, status remained excellent (NYHA I, 6MWD 442–516 m, MLHFQ 0–1), with normal renal/hepatic function and trace‐to‐mild MR (LVEF 59%–67%). At 8–9 years, the patient stayed in NYHA I with high capacity (6MWD 552–567 m), good QoL (MLHFQ 7–17), stable NT‐proBNP (107–123 pg/mL), mildly reduced LVEF (53%–57%), preserved renal function, and no right‐sided congestion or other valve disease.

**Figure 5:**
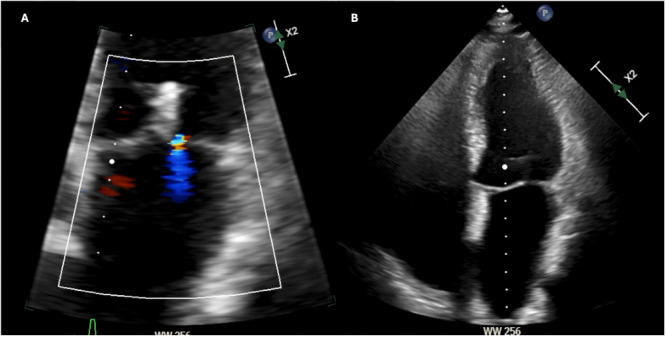
Transthoracic echocardiography at 9‐year follow‐up. Transthoracic imaging at 9‐year follow‐up showed mild residual mitral regurgitation (A) and left ventricular reverse remodeling (B). [Color figure can be viewed at wileyonlinelibrary.com]

Overall, the patient experienced sustained clinical, functional, and echocardiographic improvement over a 9‐year follow‐up period after M‐TEER, with durable reduction of MR to mild or less from 6 months onwards, reaching trace‐to‐mild (< grade I) at 6 and 7 years (Figure 5).

## 6 Discussion

The present case demonstrates the long‐term durability and clinical benefit of transcatheter edge‐to‐edge repair (M‐TEER) for severe secondary mitral regurgitation in a critically ill patient with cardiogenic shock. Previous randomized trials, such as COAPT and RESHAPE‐HF‐2 have reported favorable outcomes after M‐TEER, but long‐term follow‐up data beyond 5 years remain limited [1, 2, 3, 4]. Beyond that, both studies excluded patients presenting with cardiogenic shock [5]. In this patient, M‐TEER resulted in immediate hemodynamic stabilization and sustained symptomatic improvement over 9 years. While the clinical course observed in this case represents a particularly favorable outcome, it highlights the potential of M‐TEER to achieve meaningful stabilization and long‐term recovery in selected patients even in the setting of cardiogenic shock. Outcomes in this population may vary depending on underlying ventricular function, loading conditions, and timing of intervention, underscoring the importance of individualized patient selection.

In line with prior reports, the patient experienced substantial improvement in functional status and quality of life. NYHA functional class improved from IV at baseline to I from the fourth year onwards and remained stable thereafter. Exercise capacity, as measured by the 6‐min walk distance, increased progressively to 567 m at 9‐year follow‐up. Likewise, MLHFQ scores improved markedly, reflecting sustained quality‐of‐life benefit. These outcomes compare favorably with published long‐term registry data, which suggest durable symptomatic improvement and survival benefit in selected patients undergoing M‐TEER [4]. The recently published 2025 ESC/EACTS guidelines provide a Class I Level A recommendation for the treatment of patients with ventricular SMR and certain echocardiographic criteria which were mostly fulfilled in our patient (suitable anatomy, NYHA class of at least III, LVESD ≤ 70 mm, at least one heart failure hospitalization or elevated NT‐proBNP levels, sPAP < 70 mmHg, no severe RV dysfunction, no coronary artery disease requiring revascularization, no severe aortic or tricuspid valve disease and no hypertrophic, restrictive, or infiltrative cardiomyopathies) [6]. The only criterion which was not met was absence of stage D or advanced heart failure. However, there have been reports about the beneficial effect of TEER in patients with advanced heart failure in the absence of alternative treatment options and prolonged circulatory support [7, 8].

Regarding the underlying etiology, the clinical presentation of de novo dilated cardiomyopathy in the setting of chronic heavy alcohol consumption strongly suggests alcoholic cardiomyopathy as a contributing factor [9]. This is further supported by the marked and relatively rapid recovery of LV function, which is more consistent with a potentially reversible myocardial injury than with typical ischemic or idiopathic DCM [10].

Importantly, in our case LV function showed significant recovery, with LVEF improving from 15% at baseline to normal values (> 60%) during follow‐up. This pattern may reflect a combination of reverse remodeling after effective MR reduction and subsequent administration of guideline directed medical therapy (GDMT). Residual MR was reduced to mild‐to‐moderate at discharge and remained mild from 6‐months onwards. Importantly, GDMT could gradually be uptitrated and optimized after M‐TEER [11, 12]

Cardiorenal and cardiohepatic syndromes also improved substantially. Baseline renal dysfunction (eGFR 25 mL/min/1.73 m^2^) and hepatic impairment (bilirubin 10.4 mg/dL) normalized within the first year and remained stable over long‐term follow‐up. This observation is consistent with prior evidence that reduction of severe MR can reverse secondary end‐organ dysfunction [13]. This case emphasizes the validity of M‐TEER as a durable therapeutic option for severe MR, including in the context of cardiogenic shock, as well as the importance of regular follow‐up visits in an experienced heart valve center to ensure uptitration of GDMT to maximize functional recovery.

## 7 Conclusions

This case highlights several important aspects. First, even in the setting of cardiogenic shock and multiorgan failure, M‐TEER can provide immediate stabilization, which may translate into long‐term benefits. Second, durable reduction of MR to mild or less was associated with reverse remodeling, normalization of end‐organ function, and sustained functional recovery. Finally, this 9‐year follow‐up contributes unique evidence to the growing body of literature supporting the long‐term durability of M‐TEER.

## Funding

The authors have nothing to report.

## Conflicts of Interest

Lukas Stolz received speaker honoraria from Edwards Lifesciences. Jörg Hausleiter has received research support and speaker honoraria from Edwards Lifesciences. Michael Näbauer has received speaker fees from Abbott Vascular and Edwards Lifesciences. The other authors do not have any conflict of interest to declare.

## Data Availability

The data that support the findings of this study are available from the corresponding author upon reasonable request.
